# Some Attempts at Human Anaplasia by Means of Mucous Grafts from the Cheeks and Tongues of Rabbits and Oxen

**Published:** 1873-06

**Authors:** Houze De L’aulnoit

**Affiliations:** Lille


### Some attempts at Human Anaplasia, by means of Mucous Grafts taken from the Cheeks and Tongues of Rabbits and Oxen.

By Dr. HOUZE DE L'AULNOIT, of Lille.

Note read at the Academy of Medicine of Paris, 24th September, 1872.*

Translated from the French by MARY C. PUTNAM, M. D.

My intention in this note is to call the attention of the Academy to the results that I have obtained in transplanting on man, pieces of mucous membrane taken from the buccal cavity of the rabbit and the ox. One of the principal inconveniences of the human dermo-epidermic graft, as it has been employed by Reverdin (*Gaz. Med. de Strasbourg*, 1870; *Archives Gen. de Med.*, 1872), Ollier (*Gaz. Hebd.*, No. 8, 1872), L. Le Fort (*Gaz, Hebd.*, No. 9, 1872), Broca (Thése de M. Bercaru), Gosselin (ibid.), Demarquay (ibid.), Pollock, Nelson, Rouge, and Bercaru (Thése, Agust 7, 1872, Paris), is to cause pain to the patients by submitting them to a slight operation, without danger it is true, but disagreeable enough to be refused by some, thus obliging the surgeon to take the grafts from his own person.

I had thought, then, since the month of November, 1871, of taking them from an animal, as had already been done by the advice of Bert (*Thése sur la Graffe, Animale*), Coze (Communication to the Institute, the 28th February, 1872), who cut cutaneous grafts from a rabbit, and Debreuill, from Guinea-pigs. But in order to approach the physiological conditions of the human skin, I intended to make graft from the mucous membrane of some animal. I communicated my idea, on the 30th of April, to Professor Verneuil, and, as I mentioned the rabbit among the animals most suited to this class of experiments, he advised me to choose a rabbit raised in the country, as offering more plasticity than those brought up in our great cities.

I began my experiments on the 1st July, 1872, and I placed on several patients fourteen little mucous grafts, coming either from the cheek or the tongue of a rabbit.

Twice, moreover, I took grafts from the lingual mucous membrane of an ox, killed hardly an hour before. As the result was negative, I will pass over these two attempts in silence, and in this note only insist on the fourteen preceding experiments.

A few words on the method of operation that I followed.

Before detaching the mucous membrane, I kill the animal by holding it by the hind legs, and striking it on the back of the neck; then, by means of two sections made with scissors, I remove the skin of the cheek, and dissect, with pincers and a bistoury, from its internal face, the piece of mucous membrane that should

* GAZETTE HEBDOMAD. DE PARIS, October 11, 1872.

be applied to human tissues. The method of MM. Ollier and Reverdin, would cause less dragging on the parts, and be followed, I think, with similar results. It suffices to drag the tongue forwards, after having separated the maxillæ, in order to lift up the lingual mucous membrane, previously fastened on a piece of cork with some pins. I avoid as much as possible leaving any muscular tissue on its adherent face. These severed grafts, divided into others smaller, from five millimetres to 2-3 centimetres, have been immobilized on wounds, sometimes with bands of muslin covered with collodion at their internal face, or merely at their extremities, sometimes with cotton-wool slightly compressed by a few bands of diachylon. In some cases I tried covering them with oiled silk of very thin pieces of lead, at the same time appling a light and gentle compression.

Immobility is a condition, *sine qua non*, of success. It is impossible to insist too much on its advantages or its inconveniences, according as one employs collocion, cotton, or diachylon strips. I propose in a forthcoming memoir to discuss these various methods of treatment. Perhaps it would be possible to fasten the grafts, when they are extensive, with some points of silk suture, made with very fine needles. The strips of diachylon, with or without cotton-wool, merit the preference when their application is possible, but on condition that one may rely on the docility of the patients, and that the tissues remain motionless, at least during the first days. It is because I did not take sufficiently into account these precepts and the state of the wouds,—conditions which have only been revealed to me by experiment,—that I have only succeeded five times; that four times I have had a doubtful adhesion; and that I have failed in the other five cases.

*Conclusions.*—From these first experiments of animal anaplasty, by means of the lingual and buccal mucous membranes, I think I may deduce the following conclusions:—

1. Mucous grafts taken from the cheek or tongue of the rabbit, like the dermo-epidermic grafts from the human skin, can take root in human tissues.

2. They may be applied during the bleeding and granulating periods; never during the period of suppuration.

3. New researches are indispensable in order to learn the best method of immobilization, and the epoch at which the first dressing should be removed—epoch that I fix at the second or third day.

4. At the moment that vascular adhesions are formed, the epidermis separates, the dermis inflames, softens, disintegrates, loses its physiological properties, and becomes a true pathological tissue, having some resemblance to cicatricial tissue, but without the retractility. Microscopical examination indeed shows destruction of the glandular system.

5. Adhesion, in my fourteen experiments, only took place five times, and four times the result was doubtful.

6. Two attempts with the tongue of an ox were negative, on account of the condition of the wound or nature of the dressing.

These researches, although extremely incomplete, will induce, I hope, physiologists and surgeons to have recourse to the mucous membrane of an animal; but I think that, in order to prevent reabsorption, it would be best to borrow as much as possible from an animal approaching the size of a man.—*Archives of Scient. and Pract. Med.*

